# Institutional Needs

**Published:** 1913-07-19

**Authors:** 


### Institutional Needs.

APENTA WATER.

As a mild aperient Apenta Water has long been regarded with favour in this country, and a testimony to its value in the tropics, more particularly in West Africa, is now announced. The Imperial Government Physician, Dr. Külz, is quoted as speaking favourably of its qualities—that it proves reliable in hot climates, and is taken readily by patients. Apenta Water as a remedy for chronic constipation has therefore special claims as a summer aperient.

